# High-Risk Apical Hypertrophic Cardiomyopathy Requiring an Implantable Cardioverter-Defibrillator: A Case Report of an Overlooked Etiology

**DOI:** 10.7759/cureus.41564

**Published:** 2023-07-08

**Authors:** Azeem Rathore, Julia C Fortier, Kai Chen, Dinesh Kadariya, John N Catanzaro

**Affiliations:** 1 Internal Medicine, University of Florida College of Medicine – Jacksonville, Jacksonville, USA; 2 Internal Medicine, University of Florida College of Medicine, Gainesville, USA; 3 Cardiology, University of Florida College of Medicine – Jacksonville, Jacksonville, USA; 4 Cardiology/Electrophysiology, University of Florida College of Medicine – Jacksonville, Jacksonville, USA

**Keywords:** icd, implantable cardioverter defibrillator, cmr, cardiac magnetic resonance imaging, ecg, electrocardiogram, aphcm, yamaguchi syndrome, apical hypertrophic cardiomyopathy

## Abstract

Apical hypertrophic cardiomyopathy is a rare variant of hypertrophic cardiomyopathy characterized by abnormal heart muscle thickening, specifically affecting the left ventricle's apex. Classically revealing both giant T-wave inversions in the precordial leads of an electrocardiogram and a spade-like configuration of the left ventricular cavity on ventriculograms, the diagnosis of the apical variant has evolved with cardiac magnetic resonance imaging. Despite being well known among East Asian populations, the diagnosis of apical hypertrophic cardiomyopathy is often underestimated and overlooked among American patients due to the non-specific nature of echocardiography. In this case report, we present the diagnosis of apical hypertrophic cardiomyopathy in a middle-aged African American male with chronic palpitations. The diagnosis was confirmed using cardiac magnetic resonance imaging, which revealed extensive myocardial fibrosis. Ultimately, the patient was treated with an implantable cardioverter-defibrillator. Our case aims to enhance the understanding and facilitate the recognition and management of apical hypertrophic cardiomyopathy, particularly among non-Asian individuals. Current challenges revolve around robust risk stratification strategies for patients at high risk for sudden cardiac death that require device therapy.

## Introduction

Apical hypertrophic cardiomyopathy (apical HCM), also known as Yamaguchi syndrome, is a rare variant of hypertrophic cardiomyopathy (HCM). It is characterized by abnormal thickening (hypertrophy) of the heart muscle, specifically affecting the apex of the left ventricle (LV) [1]. Unlike the more common form of HCM, which typically involves the septum between the ventricles, apical HCM primarily affects the apex. On a standard 12-lead electrocardiogram (ECG), apical HCM is often associated with distinct "giant" T-wave inversions in the precordial leads defined as a negative voltage of ≥1 mV (≥10 mm) [1]. Like other forms of HCM, apical HCM also manifests with a range of vague signs and symptoms, including chest pain, dyspnea, palpitations, and even sudden cardiac death (SCD) [2]. By some estimates, HCM occurs in roughly 1 in 500 persons, of which 8% are of the apical HCM variant, and it affects males more frequently than females and often during midlife [1,3]. Demographically speaking, the prevalence of apical HCM is more common among Asians, specifically among Japanese cohorts. In one comparative study, the prevalence of apical HCM was found to be 15.2% among Japanese patients and only 1.4% among American patients [4]. These findings highlight not only the geographical variation in the distribution of apical HCM but also a potential genetic and/or environmental influence on its occurrence. While it occurs less frequently than other variants, apical HCM warrants heightened attention for prompt recognition and management, especially given its association with other comorbid events, such as SCD, atrial fibrillation (AF), and other arrhythmias [3]. Due to vague and non-specific clinical symptoms, there is an increased reliance on cardiac imaging tools for the prompt diagnosis of apical HCM. While echocardiography is often the first tool to detect LV structural findings, such as left ventricular hypertrophy (LVH), for various cardiovascular diseases, its efficacy compared to cardiac magnetic resonance (CMR) imaging is limited in the diagnosis of apical HCM [3,5]. Indeed, CMR is a more sensitive tool that can provide high-resolution images to illustrate the "spade-like" configuration of the left ventricular cavity in end-diastole [3]. While previously thought of as a "benign" form of HCM, there has been an accumulation of gradual data based on both retrospective and case report literature that has provided further insight [6-9]. Within this context, we present a case of apical HCM in a middle-aged African American male with chronic palpitations that were evaluated by electrophysiology (EP) and ultimately treated with an implantable cardioverter-defibrillator (ICD) due to concerning CMR findings. We aim to highlight both the presence of apical HCM among African Americans and also risk stratification to guide decision-making for device therapy.

## Case presentation

**Figure 1:**
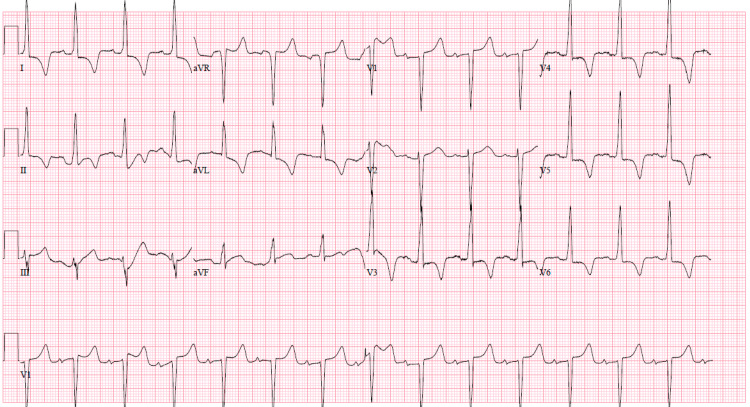
ECG depicting normal sinus rhythm of 84 bpm with significant LVH and deep T-wave inversions in leads I, II, aVF, and V3-V6 with a prolonged QT (512 ms). ECG: electrocardiogram; bpm: beats per minute; LVH: left ventricular hypertrophy

A 51-year-old African American male with a medical history of long-standing hypertension, hyperlipidemia, and chronic kidney disease was referred to cardiology by his primary care physician after an abnormal transthoracic echocardiogram (TTE) that suggested a possible infiltrative process (Video 1). The patient reported a history of intermittent palpitations that had been occurring over the past few years lasting between 5 to 10 minutes. He also endorsed occasional concomitant substernal, non-radiating chest pain with exertion that was not relieved by any medications but improved with rest. He additionally reported occasional dyspnea on exertion during these episodes too. He denied any pre-syncope or syncopal events. His initial vitals were unrevealing with a blood pressure of 137/69 mmHg, a heart rate of 60 beats per minute, a respiratory rate of 18 breaths per minute, and an oxygen saturation of 98% on ambient air. His physical examination was also unremarkable other than a notable systolic ejection murmur. An ECG was performed that was revealing for significant LVH with deep T-wave inversions in leads I, II, aVF, and V_3_-V_6_ (Figure 1).

**Video 1:** Parasternal long-axis showing severe, concentric LVH in the mid-to-apical segments with an EF >70% and a mid-intracavitary gradient of 14 mmHg at rest with a maximum intracavitary gradient of 30 mmHg; pseudonormal LV filling pattern and abnormal GLS indicating myocardial dysfunction. TTE: transthoracic echocardiogram; LVH: left ventricular hypertrophy; EF: ejection fraction; GLS: global longitudinal strain

**Figure 2:**
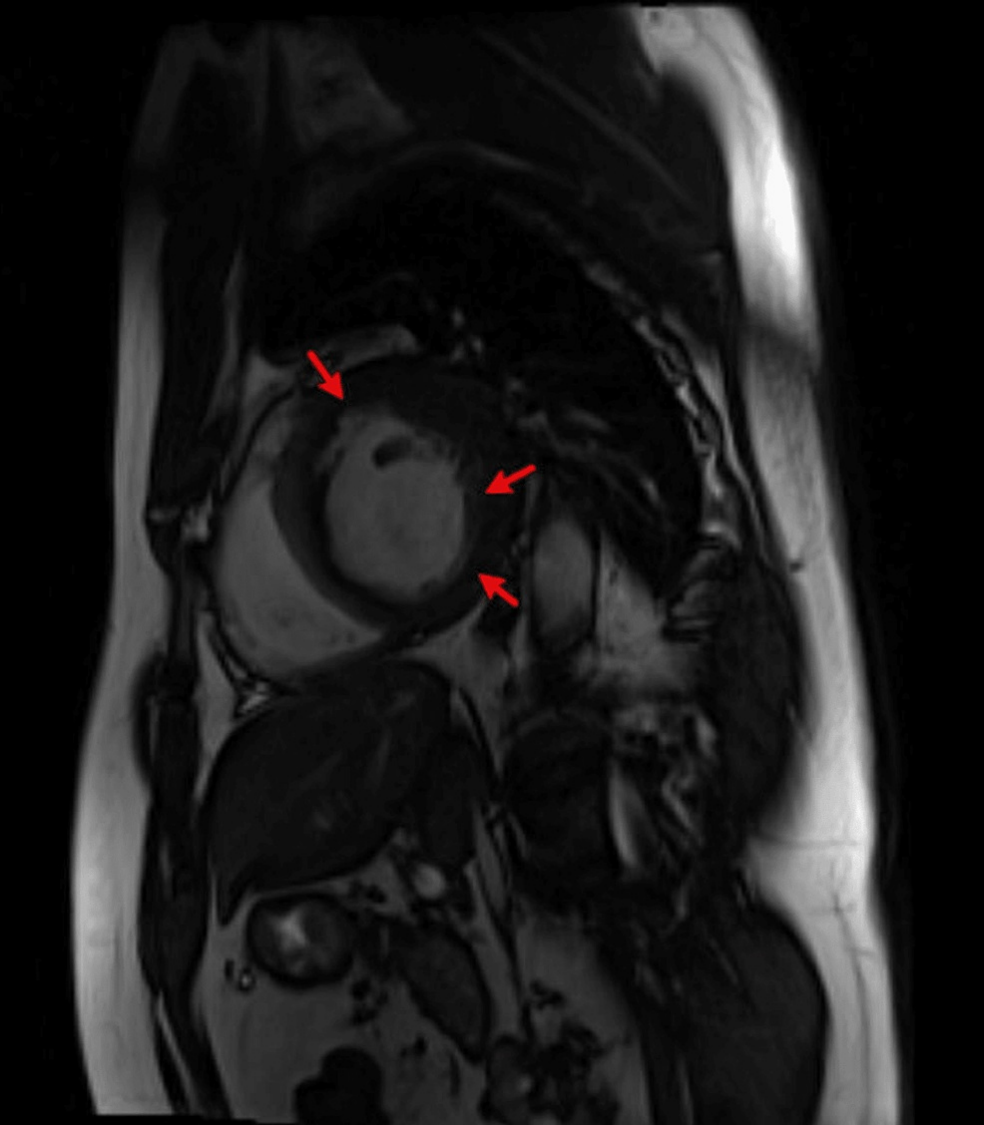
LGE and scar with resting perfusion abnormalities within the thickened mid-to-apex myocardium observed circumferentially on CMR. LGE: late gadolinium enhancement; CMR: cardiac magnetic resonance

The patient underwent cardiac event monitoring that showed sinus tachycardia without any atrial or ventricular arrhythmias. A follow-up CMR study revealed a spade-like configuration of the LV cavity during end-diastole, a ratio of an apical-to-basal left ventricular thickness of 1.7 at end-diastole, and a hyperdynamic LV ejection fraction of 72% (Video 2). Additionally, extensive mid-myocardial late gadolinium enhancement (LGE) extending from the mid-to-apex circumferentially was noted as well (Figure 2). With his clinical history, ECG reading, and imaging findings the patient was diagnosed with apical HCM. He was started on carvedilol 25 mg twice daily and later underwent placement of an ICD. Over the three years since diagnosis, the patient has been asymptomatic while compliant with beta-blocker therapy. A repeat TTE at three years was relatively stable without any worsening features compared to the previous one. Follow-up serial ECGs have also been stable in sinus rhythm, LVH, and with persistent giant T-wave inversions. No repeat CMR has been done and no genetic screening was pursued either.

**Video 2:** Characteristic spade-like configuration observed in the LV in end-diastole consistent with apical HCM seen on CMR. LV: left ventricle; HCM: hypertrophic cardiomyopathy; CMR: cardiac magnetic resonance

## Discussion

LVH is a common imaging finding in everyday clinical practice, observed in various conditions such as arterial hypertension, aortic stenosis, HCM, cardiac amyloidosis, Fabry disease, or Friedreich's ataxia [5]. LVH has been associated with adverse outcomes and the development of heart failure, ischemic heart disease, ventricular arrhythmias, and stroke. While echocardiography plays a significant role in identifying LVH and its underlying etiologies, false negatives occur. One Chinese retrospective study found that up to 31.7% of echocardiograms initially failed to diagnose apical HCM that would later be identified on CMR [6]. Indeed, CMR is the preferred imaging modality for assessing these phenotypes as it provides high-resolution images with complete coverage of LV morphology, including the apex [1,6]. Additionally, the diagnostic criteria for apical HCM have evolved, and currently, it is based on parameters such as LVH predominance in the LV apex and specific wall thickness ratios [1, 2]. Previously, a diagnosis of apical HCM required a collection of findings including marked apical obliteration via left ventriculography showcasing the unique "spade‐like configuration" as well as the ECG findings of giant negative precordial T‐waves with a voltage of >10 mm [1]. However, with advances in imaging, namely CMR, apical HCM diagnosis has evolved to include LVH predominance in the LV apex with a wall thickness in the apex ≥15 mm and a ratio of maximal apical to posterior wall thickness ≥1.5 based on echocardiography or CMR [1]. As for ECG findings, while the deep negative T waves are characteristic, they are no longer mandatory for diagnosis.

There have been a few attempts to classify apical HCM into different morphologies of pure focal, pure diffuse, and mixed phenotypes to possibly risk stratify the variants; however, their clinical relevance and acceptance have been limited [2,5]. In terms of complications, the authors of one study were interested in examining the impact of AF on the clinical progression of apical HCM [7]. The findings suggested that AF in apical HCM patients was associated with an increased risk of adverse cardiovascular events, including heart failure and thromboembolic complications, highlighting the importance of managing AF in apical HCM patients. The secondary objectives of such studies have been to assess different risk stratification models since there remain many unknowns regarding apical HCM. As previously mentioned, apical HCM has historically been viewed as a benign entity. In a prominent study of North American patients, the authors investigated the long-term outcome of patients with apical HCM over a mean follow-up period of 8.6 years and found an overall mortality rate of 10.5% [8]. The incidence of SCD accounted for 3.1% of the deaths, indicating that while apical HCM lacks the obstructive mechanism behind SCD in HCM fatal complications can occur, such as from apical aneurysm or cardiac arrest. Further, one-third of the patients experienced serious cardiovascular complications, such as myocardial infarction and arrhythmias. The authors of another study investigated the long-term prognosis of patients with apical HCM finding that the overall survival rate at 20 years was 90%, with an annual mortality rate of 0.6% [9]. Similar to previous outcome studies, the risk of cardiovascular mortality was low, suggesting a relatively favorable prognosis for individuals with apical HCM.

The management strategies for apical HCM remain complex and continue to evolve. As with HCM, mainstay medical therapy for apical HCM includes beta-blockers with the goal of reducing the prevalence of non-sustained ventricular arrhythmias [2]. In cases where beta-blockers are ineffective or not well tolerated, non-dihydropyridine calcium channel blockers (such as verapamil or diltiazem) are recommended. Other areas of active investigation include the role of alcohol septal ablation, myomectomy, and the use of LV assist devices of which all three appear to have limited efficacy in apical HCM compared to primary HCM [1,2]. Once the diagnosis of apical HCM was strongly suspected in our patient, he was started on beta-blocker therapy. While prophylactic use of ICDs to prevent SCD is widely practiced in patients with primary HCM, the decision to pursue ICD insertion in patients with apical HCM is less clear [1,7,8]. Not only are there no trials or predictive models to guide ICD therapy for apical HCM, but patients tend to score negatively for a family history of SCD, and thus there is concern that risk may be underestimated in these patients. Regardless, patients with apical HCM generally undergo comprehensive SCD risk stratification based on conventional risk factors associated with primary HCM (Table 1). Our patient not only lacked conventional risk factors but did not meet a class I recommendation for ICD placement either [1,2]. While not supported by a large body of evidence, there are other potential risk factors to help risk stratification when the concern for SCD is uncertain, including the presence of an apical aneurysm, AF, myocardial bridging, obstructive sleep apnea, or LGE by CMR [2].

**Table 1:** Clinical profile currently used to identify those patients at the highest risk for SCD who are potential candidates for ICD [2]. SCD: sudden cardiac death; ICD: implantable cardioverter-defibrillator

Conventional Risk Factors for SCD
History of cardiac arrest
Spontaneous sustained ventricular tachycardia
Family history of SCD
Unexplained syncope
Left ventricular thickness ≥30 mm
Non-sustained ventricular tachycardia
Left ventricular outflow tract obstruction gradient ≥30 mmHg

While the 2020 AHA/ACC guidelines list LGE by CMR as a class IIb recommendation for ICD implantation in primary HCM, there is no dialogue on device therapy in the apical subtype [10]. In fact, the extent or presence of LGE is not even included in the European Society of Cardiology HCM risk‐stratification algorithm [11]. However, some clinicians have argued that LGE should be considered a novel high‐risk marker to guide device insertion [1]. As for our patient, we believed the presence of LGE >15% of total LV mass raised the risk for arrhythmogenic SCD and thus performed ICD placement. Indeed, as suggested by both cohort studies and case report literature, the risk of SCD in apical HCM is not negligible [12-15]. A recent retrospective study found that patients with apical HCM had an estimated SCD rate of 2.7% per year in their sample population, of which a personal history of unexplained syncope, non-sustained ventricular tachycardia (VT), and LGE by CMR were common risk factors noted [12]. They concluded that high-risk patients with apical HCM should be considered for ICD implantation in the same manner as the septal type of HCM. In terms of outcome, the prognosis of apical HCM in patients with ICD has been shown to also be promising. In another retrospective study of patients in an ICD clinic, the authors found over a mean follow-up period of 7.2 years that the survival rate among patients with apical HCM with an ICD was high at 95.8%, with a low incidence rate of major adverse cardiac events at 2.3% per year highlighting the effectiveness of ICD therapy in improving prognosis and reducing the risk of SCD [13].

The risk of not pursuing device therapy in apical HCM has been highlighted in case report literature. In one case, a patient experienced ventricular fibrillation (VF) cardiac arrest and subsequently received an ICD for secondary prevention [14]. In that case, the patient denied a family history of recurrent syncope or unexplained cardiac death but reported unspecified cardiac hypertrophy, arrhythmia in one brother, and coronary artery disease in their mother. The diagnosis was made via ventriculogram as the TTE was unremarkable, and CMR was not performed. In another case report, an African American patient had multiple admissions presumed to be primarily due to hypertensive heart disease. However, upon closer review of his ECGs and echocardiography, the authors suspected a diagnosis of apical HCM was suspected [15]. Subsequent CMR confirmed the diagnosis, revealing near-complete cavity obliteration of the LV apex and significant LGE. While the patient was medically optimized with beta-blocker therapy, the care teams did not discuss pursuing device therapy. Unfortunately, the patient died six months later due to presumed SCD. In the absence of robust evidence-based studies, clinicians will have to rely on both retrospective studies and case reports to help further understand the risk profile of SCD in patients with apical HCM. Our case report provides an instance whereby primary prevention with ICD implantation was pursued in the setting of extensive myocardial fibrosis in a patient with apical HCM who on follow-up two years later has not experienced any significant cardiovascular event or worsening in his symptoms.

## Conclusions

We present the case of a 51-year-old African American male with apical hypertrophic cardiomyopathy managed successfully with beta-blockers and ICD placement. He presented with nonspecific symptoms including palpitations, angina, and dyspnea. Large inverse T waves in the precordial leads on ECG hinted toward the diagnosis of apical HCM, which was confirmed on CMR by demonstration of a spade-like configuration of the LV in end-diastole. While our patient did not meet any class I recommendations for ICD placement, the extensive LGE >15% portended a high risk of SCD and thus ICD implantation was performed. Cardiologists should maintain a broad differential diagnosis to consider apical HCM among African Americans in the presence of giant T-wave inversions and abnormal echocardiogram findings.
